# Microwaves from GSM Mobile Telephones Affect 53BP1 and γ-H2AX Foci in Human Lymphocytes from Hypersensitive and Healthy Persons

**DOI:** 10.1289/ehp.7561

**Published:** 2005-04-28

**Authors:** Eva Markovà, Lena Hillert, Lars Malmgren, Bertil R. R. Persson, Igor Y. Belyaev

**Affiliations:** 1 Department of Genetics, Microbiology and Toxicology, Stockholm University, Stockholm, Sweden; 2 Laboratory of Molecular Genetics, Cancer Research Institute, Bratislava, Slovak Republic; 3 Occupational and Environmental Health, Stockholm County Council, Stockholm, Sweden; 4 Department of Public Health Sciences, Division of Occupational Medicine, Karolinska Institutet, Stockholm, Sweden; 5 MAX-lab, Lund University, Lund, Sweden; 6 Department of Medical Radiation Physics, Lund University Hospital, Lund, Sweden; 7 Laboratory of Radiobiology, General Physics Institute, Russian Academy of Science, Moscow, Russia

**Keywords:** 53BP1 and γ-H2AX foci, chromatin, DNA double-strand breaks, hypersensitivity to electromagnetic fields, stress response

## Abstract

The data on biologic effects of nonthermal microwaves (MWs) from mobile telephones are diverse, and these effects are presently ignored by safety standards of the International Commission for Non-Ionizing Radiation Protection (ICNIRP). In the present study, we investigated effects of MWs of Global System for Mobile Communication (GSM) at different carrier frequencies on human lymphocytes from healthy persons and from persons reporting hypersensitivity to electromagnetic fields (EMFs). We measured the changes in chromatin conformation, which are indicative of stress response and genotoxic effects, by the method of anomalous viscosity time dependence, and we analyzed tumor suppressor p53-binding protein 1 (53BP1) and phosphorylated histone H2AX (γ-H2AX), which have been shown to colocalize in distinct foci with DNA double-strand breaks (DSBs), using immunofluorescence confocal laser microscopy. We found that MWs from GSM mobile telephones affect chromatin conformation and 53BP1/γ-H2AX foci similar to heat shock. For the first time, we report here that effects of MWs from mobile telephones on human lymphocytes are dependent on carrier frequency. On average, the same response was observed in lymphocytes from hypersensitive and healthy subjects.

The growing public concerns about possible effects of microwave (MW) exposure from mobile telephones have been discussed in many countries because of increasing use of wireless communication systems. Two groups have reported that increased incidence of brain tumors and acoustic neuroma is correlated with exposure to mobile telephone MWs depending on duration of mobile telephone use (Hardell et al. 2003; Lonn et al. 2004). Negative findings were also reported by other groups, but the results of these epidemiologic studies are not directly comparable because of methodologic and other differences, as has recently been reviewed (Kundi et al. 2004). The intensity levels of exposure to MWs from mobile telephones are lower than the standards adopted by the International Commission for Non-Ionizing Radiation Protection (ICNIRP 1998). These standards are based on the thermal effects of MWs resulting in heating of exposed tissues or cells. There is also evidence for nonthermal effects of MWs that suggests a possible relationship between nonthermal MW exposure and both permeability of the brain–blood barrier (Persson et al. 1997) and stress response (de Pomerai et al. 2000). Recent studies have described neuronal damage in the brains of exposed rats (Ilhan et al. 2004; Salford et al. 2003). In other studies, no effects of nonthermal MWs were observed (Meltz 2003). However, experimental data suggested that MW effects occur only under specific parameters of exposure, depending on several physical parameters and biologic variables (Adey 1999; Belyaev et al. 2000; Binhi 2002; Blackman et al. 1989). Dependence of the MW effects on several physical parameters, including frequency, polarization, and modulation, and also several biologic variables could explain various outcomes of studies with nonthermal MWs (Belyaev et al. 2000).

MWs under specific conditions of exposure induce DNA strand breaks in rat brain cells as measured by single-cell electrophoresis (Lai and Singh 1996, 1997). The mechanisms of this effect are not understood, but they could be related to induced changes in the interaction of DNA with proteins, rather than DNA damage (Belyaev et al. 1999).

Several proteins, such as the tumor suppressor p53-binding protein 1 (53BP1) and phosphorylated H2AX (γ-H2AX), have been shown to produce discrete intranuclear foci, which are believed to colocalize with DNA double-strand breaks (DSBs) providing a scaffold structure for DSB repair (DiTullio et al. 2002; Schultz et al. 2000; Sedelnikova et al. 2002). According to the current model, this scaffold functions by recruiting proteins involved in the repair of DSBs (Fernandez-Capetillo et al. 2002; Iwabuchi et al. 2003; Kao et al. 2003). The scaffold is organized within a megabase-size chromatin domain around an actual DSB regardless of the type of repair that is involved (Paull et al. 2000). In an analysis of the 53BP1 foci in human lymphocytes after exposure to MWs from mobile telephones using the Global System for Mobile Communication (GSM) standard at 915 MHz, we did not find induction of 53BP1 foci (Belyaev et al. 2005). In contrast, we found that MWs similar to heat shock induced significant reduction in the background level of 53BP1 foci (Belyaev et al. 2005). In the present study, we analyzed the γ-H2AX protein in addition to the 53BP1 protein. We also applied the method of anomalous viscosity time dependence (AVTD) that is sensitive to various genotoxic effects (Belyaev et al. 1999, 2001).

So-called hypersensitivity to electromagnetic fields (EMFs) is a fairly new phenomenon, and etiology of the hypersensitivity to EMFs is not yet known. There are several symptoms that people experience in proximity to different sources of EMFs, such as video display terminals of personal computers, electrical appliances, or mobile telephones. The symptoms are not specific for this illness, and there is no known pathophysiologic marker or diagnostic test (Hillert et al. 1999).

There is a substantial lack of knowledge in the biophysical modeling of MW-induced nonthermal biologic effects. Resonance-like interactions of MWs with such targets as cellular membranes, chromosomal DNA, and ions in protein cavities have been proposed (Adey 1999; Belyaev et al. 1992a; Binhi 2002; Ismailov 1987).

Among other dependencies, dependence of nonthermal effects of MWs on frequency has been reported (Belyaev et al. 2000; Pakhomov et al. 1998). In a recent study of nonthermal effects of GSM MWs at various frequencies on the conformation of chromatin in human lymphocytes, Sarimov et al. (2004) found that MWs from GSM mobile telephones affect chromatin conformation in human normal and transformed lymphocytes at specific frequencies, 905 MHz and 915 MHz being most effective. The observed MW effects depended upon the initial state of chromatin as measured before exposure and were similar to stress responses induced by heating (Sarimov et al. 2004). In the present study, we analyzed the effects of MWs at different frequencies on chromatin conformation and 53BP1 and γ-H2AX foci in lymphocytes from healthy and hypersensitive subjects.

## Materials and Methods

### Subjects and blood samples.

Blood samples from five healthy subjects and five patients reporting hypersensitivity to EMFs were obtained at the Department of Occupational and Environmental Health, Stockholm County Council, Sweden. The group of hypersensitive persons was selected on the basis of self-reported hypersensitivity to EMFs and characterized regarding symptom profile, triggering factors, exposure–time relationships, and avoidance behavior (Hillert et al. 1999). The group reporting hypersensitivity to EMFs consisted of five men 32–60 years of age (Table 1). Control healthy subjects were matched by age (± 5 years) and sex (Table 1). All hypersensitive persons and controls were employed or students. None of the participants were smokers, and no subject was on any regular medication. All hypersensitive subjects reported that their symptoms were triggered by electrical devices that were not sources of light. Four of the participants reported that mobile telephones also triggered symptoms. The fifth subject did not use a mobile telephone and consequently did not know if this exposure triggered symptoms. In all pairs, the hypersensitive person scored higher than the matched control in the questionnaire on symptoms (29 symptoms scored for frequency and severity; maximum score, 232) (Hillert et al. 1998). In all persons reporting hypersensitivity to EMFs, neurovegetative symptoms such as headache, fatigue, and difficulties concentrating were more pronounced than skin symptoms. The mean scores per person for neurovegetative symptoms were 33 in the hypersensitive group and 1.2 in the control group. The corresponding scores for skin symptoms in the face and upper chest were 10 and 0.4, respectively. In all cases of reported hypersensitivity, the subjects reported experiencing symptoms 24 hr after exposure to a reported triggering factor, in most cases within 1 hr. All patients reported that they tried to avoid triggering factors.

Fresh blood samples from hypersensitive persons and from matched controls were coded and all data were analyzed blind. Ethical permissions were obtained from the Ethics Committee of the Karolinska Institutet, Stockholm, Sweden. All subjects volunteered for the study.

### Chemicals and reagents.

We obtained reagent grade chemicals from Sigma (St. Louis, MO, USA) and Merck KgaA (Darmstadt, Germany). We purchased double cytoslides coated with polylysine and cytoslide chambers from ThermoShandon (Pittsburg, PA, USA). Anti-53BP1 antibody (monoclonal mouse) was kindly provided by T. Halazonetis (Wistar Institute; University of Pennsylvania, Philadelphia, PA, USA). The antibody recognizes the C-terminal domain of the protein that corresponds to the BRCT (BRCA-1 C-terminal) domains. Anti-γ-H2AX (monoclonal rabbit) was purchased from Trevigen-BioSite (Täby, Sweden).

### Cells.

Lymphocytes were isolated from peripheral blood by density gradient centrifugation in Ficoll-Paque (Pharmacia LKB, Uppsala, Sweden) according to the manufacturer’s instructions. The cells were transferred to basal medium [BM: RPMI 1640 medium supplemented with 10% fetal bovine serum (FBS), 2 mM l-glutamine, 50 IU/mL penicillin, and 50 μg/mL streptomycin (ICN Pharmaceuticals, Inc., Costa Mesa, CA, USA)] and incubated at 5% CO_2_ and 37°C in a humidified incubator. Adherent monocytes were removed by overnight incubation of the cell suspension in Falcon culture flasks (Becton Dickinson, Franklin Lakes, NJ, USA) at a cell density of 3 × 10^6^ cells/mL in a volume of 10–40 mL. After this incubation, the cells remaining in suspension were collected by centrifugation. The cell density was adjusted to approximately 2 × 10^6^ cells/mL in fresh BM, and the lymphocytes were pre-incubated for 2 hr at 37°C before exposure. The viability of cells was always > 98% as measured with a trypan blue exclusion assay.

### Cell exposure.

In five independent experiments, coded samples from hypersensitive subjects and matched control subjects were exposed simultaneously. All exposures were performed for 1 hr at 37°C in a humidified CO_2_ incubator, in 14 mL round-bottom tubes (Falcon), 2.5 mL cell suspension per tube, 2 × 10^6^ cells/mL. Lymphocytes were exposed to MWs using a GSM900 test mobile telephone (model GF337; Ericsson, Stockholm, Sweden) as previously described (Belyaev et al. 2005; Sarimov et al. 2004). Briefly, the output of the telephone was connected by the coaxial cable to a transverse electromagnetic transmission line (TEM) cell. The 124 different channels/frequencies that are used in GSM900 mobile communication differ by 0.2 MHz in the frequency range between 890 and 915 MHz. We used channels 74 and 124 with frequencies of 905 and 915 MHz, respectively. The signal included standard GSM modulations. No voice modulation was applied, and discontinuous transmission mode was off during all exposures. GSM signal is produced as 577 μsec pulses (time slots), with an interpulse waiting time of 4,039 μsec (seven time slots). The power was kept constant during exposures, at 2 W (33dBm > 1 mW) in pulse, as monitored online using a power meter (Bird 43; Bird Electronic Corporation, Cleveland, OH, USA). The specific absorption rate (SAR) was determined by measurement and calculation. We measured transmitted and reflected power using a power meter (Hewlett-Packard 435A; Hewlett-Packard Company, Palo Alto, CA, USA) and a coaxial directional coupler (Narda 3001-20; Narda, Hauppage, NY, USA). A signal generator (Agilent 8648C; Agilent, Santa Rosa, CA, USA) connected to a power amplifier (Mini-circuits ZHL-2-8-N; Mini-circuits, Brooklyn, NY, USA) was used. The SAR, calculated from the absorbed power and the mass of the sample, was 37 mW/kg. Good correlation between these measurements and calculations using the finite different time domain (FDTD) method has been confirmed (Sarimov et al. 2004). Because of nonequal distribution of SAR through the exposed volume, the minimal and maximal FDTD-derived SARs were 2.5-fold lower and 3.3-fold higher, respectively, compared with the mean values. All these SAR values were well below thermal effects. Temperature was measured in the MW-exposed samples before and after exposure with a precision of 0.1°C. No changes in temperature were induced during exposures.

At the place of exposure, static magnetic field was 18 ± 2 μT as measured by means of a magnetometer (Sam3, Dowty Electronics Ltd., Cannock, UK) and background extremely low-frequency magnetic field was not more than 200 nT, root mean square, as measured with a three-dimensional microteslameter (Field dosimeter 3, Combinova, Bromma, Sweden).

In each experiment, the cells from the same blood samples were exposed in the same TEM cell to MWs at 915 MHz and 905 MHz and sham-exposed with MWs off. The cells were exposed in sequence, and the order of exposure was randomized. Heat treatment in a water bath, at 41°C and 43°C, was used as a positive control for stress responses. As a positive control for genotoxic effect, the cells were irradiated with ^137^Cs γ-rays, at 3 Gy, using a Gammacell 1000 source (Atomic Energy of Canada Limited, Ottawa, Canada). The dose rate was 10.6 Gy/min.

### AVTD measurements.

We studied the conformation of chromatin by the method of AVTD. Cell lysis was performed immediately after exposure as previously described (Belyaev et al. 1999). Briefly, lymphocytes were lysed in polyallomer centrifuge tubes (14 mm; Beckman, Fullerton, CA, USA) by addition of 3.1 mL lysis solution (0.25 M Na_2_EDTA, 2% wt/vol sarcosyl, 10 mM Tris-base, pH 7.4) to 0.1 mL cell suspension. The lysates were prepared in triplicate and kept at 23°C for 4 hr in darkness before AVTD measurements. The AVTDs were measured at a shear rate of 5.6/sec and shear stress of 0.007 N/m^2^. Normalized relative viscosity (NRV) was used to characterize condensation of chromatin (Belyaev et al. 1999).

### Immunostaining and foci analysis.

Immediately after exposure, the cells were placed on ice for 1 hr to prevent the repair of eventual DSBs. Cytoslide samples were prepared by using cytospin centrifugation according to the manufacturer’s instructions (ThermoShandon, Pittsburgh, PA, USA). The immunostaining was performed according to Schultz et al. (2000), with some modifications. Cells were fixed in cold 3% paraformaldehyde in phosphate-buffered saline (PBS; pH 7.4), permeabilized with cold 0.2% Triton X-100 in PBS (for 15 and 10 min, respectively), stained with primary antibody 53BP1 (1:20) and γ-H2AX (1:100) prepared in 2% FBS in PBS for 1 hr, followed by three washes in cold PBS, and incubated for 1 hr with secondary Hexo goat anti-mouse IgG (H + L) antibody conjugated with Alexa fluor 488 (Molecular Probes, Inc., Eugene, OR, USA) together with Zymax goat anti-rabbit IgG Cy3 conjugate (Zymed, San Francisco, CA, USA), both in 2% FBS and in 1:200 dilution, followed by three washes in cold PBS. After 20 min DNA staining in ToPro-3-iodide (Molecular Probes; 10 μM in PBS, prepared from 1 mM stock solution in dimethyl sulfoxide) and 5 min of washing in PBS, cytoslides were mounted with equilibration solution and antifade reagent (SlowFade Light Antifade Kit; Molecular Probes) and sealed with coverslips. The images were recorded using a confocal laser scanning microscope Zeiss Axiovert 100M (Carl Zeiss Microscopy, Jena, Germany) from 5–10 fields of vision that were randomly selected from two slides per treatment condition. Through focus, maximum projection images were acquired from optical sections 1.00 μm apart and with a section thickness of 2.00 μm in the *z*-axis. Resolutions in *x*- and *y*-axes were 0.20 μm. Seven optical sections were usually acquired for each field of vision, and a final image was obtained by projection of all sections onto one plane. The foci were counted in the cells from these final images using LSM 510 software (Carl Zeiss Microscopy). For each experimental condition, we analyzed 300–600 cells. All images were analyzed blind regarding exposure parameters.

### Statistical analysis.

We set the statistical power to 0.80 based on previously obtained data on effects of GSM MWs on human lymphocytes (Belyaev et al. 2005; Sarimov et al. 2004). We analyzed data using the Mann-Whitney *U*-test, Kruskal-Wallis test, or the Wilcoxon matched-pairs signed-rank test. A correlation analysis was performed using Spearman rank order correlation test. Results were considered as significantly different at *p* < 0.05.

## Results

### Viability.

The viability of unexposed cells as measured by the trypan blue exclusion assay varied between normal and hypersensitive subjects in the range of 0.01–2%. We found no statistical difference in the levels of viability between these groups.

### Chromatin conformation.

We observed a statistically significant decrease in AVTDs corresponding to chromatin condensation in cells of 5 subjects (subjects 301, 302, 406, 606, and 607; Table 2) of 10 at the frequency of 915 MHz (*p* < 0.05, Mann-Whitney *U*-test; Table 2). In contrast, only in cells from subject 403, we observed a significant increase in AVTDs that corresponds to decondensation of chromatin after 915 MHz. MWs at 905 MHz resulted in either significant condensation (subject 607), or decondensation (subject 403), or no effects (Table 2). These data suggested that effects of MWs might be frequency dependent and that differing responses might be observed in cells from different individuals. Similar interindividual variability was observed in response to the heat shock, especially at 43°C, where two subjects responded by condensation (subjects 406 and 707) and two by decondensation (subjects 302 and 403). We found no statistically significant differences between the effects on chromatin conformation in cells from controls and hypersensitive groups as measured after either MW exposures or heat shock (*p* > 0.05, Wilcoxon matched-pairs signed-rank test). The data pooled from all subjects, normal and hypersensitive, were analyzed for each treatment condition. The analysis of these pooled data showed a statistically significant effect of MW exposure at 915 MHz (*p* < 0.0223, Mann-Whitney *U*-test).

### Immunostaining.

**Figure 1:**
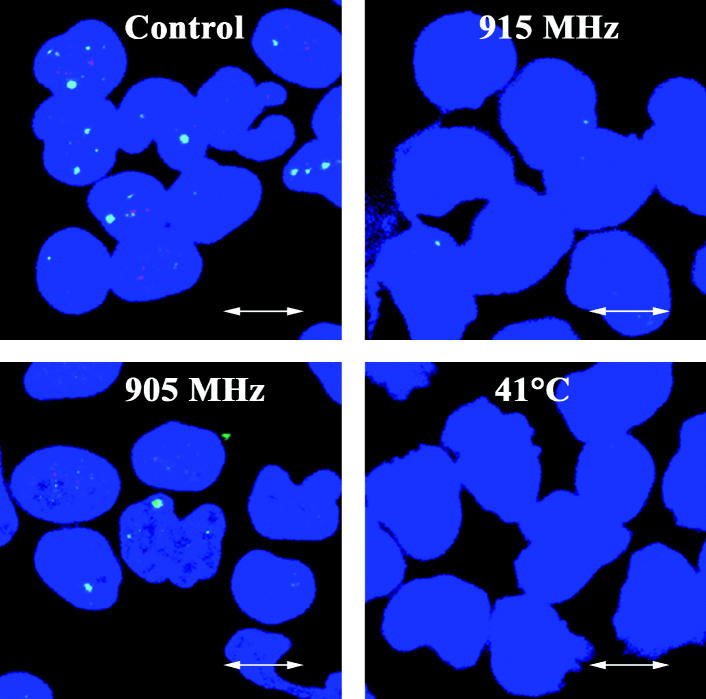
Images of fixed human lymphocytes (counterstained blue with ToPro-3-iodide) showing 53BP1 foci (stained green with Alexa fluor 488) and γ-H2AX foci (stained red with Cy3) as revealed by immunostaining and confocal laser microscopy of cells from subject 501. Significantly fewer foci were observed after 1 hr exposure to 915 MHz and heat shock (41°C) than in control cells. Exposure to 905 MHz resulted in a statistically significant increase in the number of 53BP1 foci in cells from this subject (Table 3). Bar = 10 μm.

**Figure 2:**
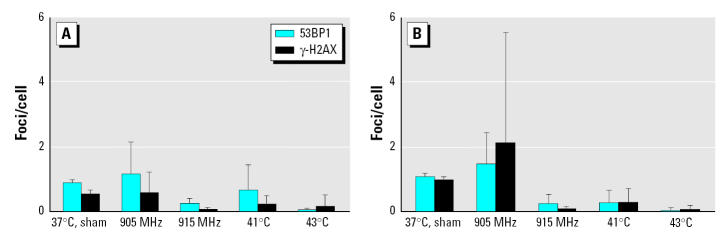
53BP1 and γ-H2AX foci in human lymphocytes of matched controls (A; n = 5) and hypersensitive subjects (B; n = 5) after exposure to 905 MHz, 915 MHz, and heat shock at 41°C and 43°C, as measured by immunostaining and confocal laser microscopy after 1 hr of treatment. Values shown are mean and SD for amounts of foci per cell from five subjects. Similar reduction of foci level was seen after 915 MHz exposure and after heat shock. Exposure to 905 MHz led to either reduction or induction of foci dependent on subject, resulting in larger SDs for this treatment compared with 915 MHz.

Our 53BP1/γ-H2AX foci analysis included a positive control with 3 Gy γ-rays. We observed a significant increase in the number of foci 1 hr after irradiation (data not shown). In contrast, neither cells from control subjects nor cells from hypersensitive subjects responded to 915 MHz by induction of foci (Table 3). We observed a distinct MW-induced reduction in the level of 53BP1/γ-H2AX foci in cells from both control and hypersensitive subjects in response to 915 MHz (Figure 1, Tables 3 and 4). Very similar reductions in 53BP1/γ-H2AX foci were observed in lymphocytes from control and hypersensitive subjects in response to heat shock at 41°C and 43°C (Tables 3 and 4, Figure 2A,B). The response to 905 MHz was not consistent among subjects, and either increase, decrease, or no effect was observed in the number of foci, dependent on the subject (Tables 3 and 4).

For each subject, we verified the hypothesis that MW exposure affects formation of 53BP1 and γ-H2AX foci. For this purpose, we compared effects of MW exposures with sham (multiple comparisons of sham, 905 MHz, and 915 MHz) using Kruskal-Wallis analysis of variance (ANOVA) by ranks. This comparison showed that MWs affected both 53BP1 and γ-H2AX foci in cells from each tested person (Table 5).

We next verified the hypothesis that the effect of MW exposure was frequency dependent by comparing MW effects at 905 MHz and 915 MHz for cells from each subject by the Mann-Whitney *U*-test. This comparison showed that MW effects on 53BP1 foci depended on frequency in cells from nine subjects (all except subject 606), and effects on γ-H2AX foci depended on frequency in cells from six subjects (all except subjects 302, 406, 607, and 708) (Table 6).

Under identical conditions of treatment, the numbers of 53BP1 and γ-H2AX foci were not significantly different between cells from matched controls and hypersensitive subjects compared using the Wilcoxon matched-pairs signed-rank test. Therefore, we pooled the data from all experiments with cells from control and hypersensitive subjects. Statistical analysis of these pooled data showed that 915 MHz exposure significantly reduced the number of 53BP1 and γ-H2AX foci in human lymphocytes (Tables 3 and 4). Despite the fact that no heating was induced by MW exposure, the reduction in the number of 53BP1 and γ-H2AX foci was larger than after heat shock at 41°C (Tables 3 and 4). In the case of γ-H2AX foci, this reduction was even larger than after heat shock at 43°C (Table 4). Importantly, the pooled effects of MWs were statistically significantly different at 915 MHz and 905 MHz for both 53BP1 and γ-H2AX foci (*p* < 0.0125 and *p* < 0.0357, respectively, Wilcoxon matched-pairs signed-rank test).

For all treatment conditions, a correlation between 53BP1 and γ-H2AX foci was observed (*R* = 0.64, *p* < 0.00001, Spearman rank-order correlations test). However, most of the 53BP1 and γ-H2AX foci did not colocalize, and the colocalization did not exceed 7%.

## Discussion

It has been previously shown that nonthermal MWs affected conformation of chromatin in Escherichia coli cells, rat thymocytes, and human lymphocytes under specific conditions of exposure (Belyaev et al. 1992b, 2000, 2002; Sarimov et al. 2004). Usually, in human lymphocytes, the AVTDs decreased transiently after exposure to nonthermal MWs as opposed to the increase in AVTDs observed immediately after genotoxic impacts, such as ionizing radiation or chemicals (Belyaev et al. 1999, 2001; Sarimov et al. 2004). Several experimental observations have suggested that the increase in the AVTDs is caused by the relaxation of DNA domains (Belyaev and Harms-Ringdahl 1996). Single-cell gel electrophoresis and halo assay have confirmed this suggestion (Belyaev et al. 1999, 2001). On the other hand, the decrease in AVTDs can be caused by either chromatin condensation or DNA fragmentation (Belyaev et al. 1999, 2001). Because no 53BP1/γ-H2AX foci were produced in response to 915 MHz, the decrease in the normalized maximum relative viscosity induced by the 915 MHz exposures was likely caused by chromatin condensation. Both decrease and increase in AVTDs were induced by heat shock at 41°C and 43°C, depending on the subject (Table 2). In contrast to a previous study (Sarimov et al. 2004) in which cells were exposed to MWs at room temperature, MW exposure was performed at 37°C in the present study. Bearing in mind the previously observed dependence of MW effects on temperature (de Pomerai et al. 2000), the data from these two studies should be compared with care. The AVTD data from both studies show that MWs and heat shock result in either condensation or decondensation of chromatin in human lymphocytes dependent on the subject and the duration and temperature of treatment. We detected no heating in samples exposed to MWs; therefore, the MW effects were not caused by heating.

The analysis of 53BP1/γ-H2AX foci is a more sensitive assay compared with other available techniques to measure DSBs, such as pulsed field gel electrophoresis or neutral comet assay. Using this sensitive technique, we did not find any genotoxic effects of 915 MHz under the specific conditions of exposure employed here. In contrast, this frequency persistently decreased the level of foci. Therefore, in the present study we confirm our previous finding that exposure at 915 MHz reduces 53BP1 foci in a manner similar to heat shock, suggesting that this frequency affects cells in a manner similar to a stress factor (Belyaev et al. 2002, 2005). The duration of exposure was 2 hr in the previous study (Belyaev et al. 2005). In the present study, we show that even shorter exposure, 1 hr, produces a significant reduction in the 53BP1 level.

In contrast to 915 MHz exposures, MWs at 905 MHz could either decrease or increase the number of foci depending on the subject. Does it mean that 905 MHz exposures induce DSBs in those cases in which foci increased? The data obtained here neither exclude nor directly support such a possibility. We should also state that we do not really know the details of the subjects’ physiologic status, and therefore this may be the determining factor.

Frequency-dependent inhibition of DNA repair by nonthermal MWs has previously been found (Belyaev et al. 1992a, 1992b). The novel result of the present study is that both 53BP1 and γ-H2AX foci can be decreased similarly by heat shock and MWs from mobile telephones. We hypothesize that stress-induced chromatin condensation either reduces availability of DNA breaks to enzymes and antibodies or disrupts DNA repair machinery that involves binding of 53BP1/γ-H2AX proteins to DSBs. If repair is affected, according to the second of these hypotheses, the obtained results may have a connection to genotoxicity and cancer.

We show here for the first time that the vast majority of 53BP1 and γ-H2AX foci do not colocalize in either sham-control or MW/heat-shock–treated lymphocytes. The formation of these foci deals with phosphorylation of 53BP1/γ-H2AX proteins (DiTullio et al. 2002; Fernandez-Capetillo et al. 2002). It is thus possible that the observed effects of MW and heat shock at the level of 53BP1/γ-H2AX foci formation was due to a change in phosphorylation. Recent evidence has indicated activation of stress-induced pathways in cultivated cells in response to MWs (Leszczynski et al. 2002). Their article indicated that mobile telephone MWs activate a variety of cellular signal transduction pathways, among them the hsp27/p38MAPK stress response pathway (Leszczynski et al. 2002). Whether activation of stress response pathways relates to apoptosis, brain–blood barrier permeability, or increased cancer in humans remains to be investigated.

The comparison of pooled data obtained with all treatments did not show significant differences between the groups of controls and hypersensitive subjects. This result might be explained by the heterogeneity in groups of hypersensitive and control persons. Even if there is such a difference, it would be masked by the large individual variation between subjects, which was observed in both control and hypersensitive groups. An additional problem may be the lack of any objective criteria for selection of a study group consisting of persons that are truly either insensitive or hypersensitive to EMFs (although this has yet to be proven).

For the first time, the data obtained in the present study clearly show that MWs from GSM mobile telephones affect simultaneously the formation of 53BP1 and γ-H2AX foci in human lymphocytes as function of carrier frequency. This result obtained in lymphocytes from both healthy and hypersensitive persons is of great importance. Such frequency dependence suggests a mechanism that does not deal with thermal heating. Investigation of this mechanism and the molecular targets of the frequency-dependent effects of MWs in the frequency range of mobile communication is a fundamental problem.

Another aspect of this finding is that criteria other than “thermal,” based on SAR and power density in acute exposures, may be needed for accurate safety standards. In particular, these safety standards certainly cannot be based on data obtained at one specific frequency.

## Conclusions

Nonthermal MWs from GSM mobile telephones at lower levels than the ICNIRP safety standards affect 53BP1 and γ-H2AX foci and chromatin conformation in human lymphocytes. These effects suggest induction of stress response and/or DNA damage. For the first time, we report that mobile telephone MWs affect 53BP1 and γ-H2AX foci dependent on carrier frequency. We also show that heat shock induces similar responses. The same responses were observed in lymphocytes from healthy subjects and from subjects reporting hypersensitivity to EMFs.

## Figures and Tables

**Table 1:** Information on hypersensitive male subjects (*n* = 5) and matched controls (*n* = 5).

Subject	Age (years)	Duration of hypersensitivity (years)
301^a^	32	5
302	33	—
403^a^	33	2
406	29	—
501^a^	47	8
502	44	—
606	45	—
607^a^	45	1
707	59	—
708^a^	60	2

a Cases of reported hypersensitivity to EMFs.

**Table 2:** Relative changes in chromatin conformation in response to MWs as analyzed by the AVTD assay immediately after exposure and normalized to sham (NRV).

	905 MHz			915 MHz			41°C			43°C		
Subject	NRV	SD	*p*-Value	NRV	SD	*p*-Value	NRV	SD	*p*-Value	NRV	SD	*p*-Value
301^a^	1.08	0.15	0.6122	0.45	0.04	0.0039**	0.75	0.10	0.0937	1.02	0.11	0.8485
302	1.33	0.18	0.1374	0.39	0.08	0.0018**	0.94	0.09	0.5422	1.31	0.10	0.0314*
403^a^	1.51	0.20	0.0256*	1.77	0.21	0.0034**	1.26	0.17	0.1486	2.43	0.39	0.0105*
406	0.72	0.15	0.1338	0.66	0.02	0.0003**	0.58	0.13	0.0304*	0.49	0.02	0.0001**
501^a^	0.81	0.29	0.5851	0.96	0.23	0.8613	0.84	0.20	0.6278	1.49	0.34	0.1008
502	0.78	0.14	0.2566	0.62	0.12	0.0844	0.56	0.12	0.0618	0.92	0.15	0.6411
606	0.90	0.07	0.2282	0.71	0.06	0.0137*	—	—	—	0.67	0.12	0.0617
607^a^	0.68	0.08	0.0200*	0.83	0.05	0.0322*	—	—	—	0.90	0.09	0.3154
707	1.16	0.14	0.3193	1.12	0.06	0.0990	1.10	0.03	0.0323**	0.80	0.03	0.0025**
708^a^	0.83	0.10	0.1814	0.97	0.12	0.8313	0.83	0.23	0.5085	0.77	0.10	0.1019
All subjects	0.98	0.28	0.4812	0.85	0.40	0.0232*	0.86	0.24	0.0831	1.08	0.56	0.4812

—, Not analyzed. Lymphocytes from subjects hypersensitive (n = 5) and matched controls (n = 5) were exposed to MWs at 905 MHz or 915 MHz during 1 hr. Means of three measurements and SD are shown along with p-values (Mann-Whitney U-test.)

a Cases of reported hypersensitivity to EMFs.

* p < 0.05.

** p < 0.01.

**Table 3:** Changes in 53BP1 foci in response to 1 hr MW exposure.

	Sham		905 MHz			915 MHz			41°C			43°C		
Subject	53BP1	SD	53BP1	SD	*p*-Value	53BP1	SD	*p*-Value	53BP1	SD	*p*-Value	53BP1	SD	*p*-Value^a^
301^a^	0.95	0.73	1.54	0.65	0.0652	0.03↓	0.06	0.0099**	0.06↓	0.04	0.0013**	0.14↓	0.09	0.0030**
302	1.45	0.81	2.44↑	0.63	0.0020**	0.17↓	0.27	0.00001**	1.80	0.93	0.3460	0.08↓	0.09	0.0000**
403^a^	0.42	0.47	2.87↑	1.48	0.0002**	0.00↓	0.00	0.0015**	0.01↓	0.03	0.0372*	0.00	0.00	0.0712
406	0.62	0.13	0.28↓	0.23	0.0031**	0.08↓	0.17	0.0002**	0.06↓	0.09	0.0000**	0.04↓	0.04	0.00001**
501^a^	1.06	0.22	1.67↑	0.50	0.0028**	0.15↓	0.11	0.0007**	0.18↓	0.14	0.0000**	0.01↓	0.01	0.00001**
502	0.66	0.25	1.35↑	0.45	0.0079**	0.25	0.23	0.0556	0.24↓	0.08	0.0066**	0.01↓	0.01	0.0003**
606	0.84	0.26	0.08↓	0.07	0.0079**	0.20↓	0.14	0.0079**	—	—	—	0.00↓	0.00	0.0001**
607^a^	1.33	0.39	0.18↓	0.08	0.0079**	0.35↓	0.18	0.0079**	—	—	—	0.01↓	0.01	0.0001**
707	0.88	0.19	1.68↑	0.44	0.0228**	0.52	0.25	0.0556	0.56↓	0.08	0.0089**	0.11↓	0.09	0.0000**
708^a^	1.62	0.39	1.09↓	0.17	0.0159**	0.70↓	0.13	0.0079**	0.85↓	0.10	0.0028**	0.10↓	0.04	0.0000**
CS	0.89	0.10	1.17	0.98	0.4728	0.24↓	0.17	0.0176**	0.67	0.78	0.3235	0.05↓	0.05	0.0037**
HE	1.08	0.10	1.47	0.98	0.5575	0.25↓	0.29	0.0013**	0.28↓	0.39	0.0072**	0.05↓	0.07	0.0061**
All subjects	0.98	0.10	1.32	0.94	0.3150	0.24↓	0.22	0.0001**	0.47↓	0.61	0.0266**	0.05↓	0.05	0.00001**

—, Not analyzed. Lymphocytes from subjects hypersensitive to EMF (HE; n = 5) and matched controls (CS; n = 5) were exposed to MWs at 905 MHz and 915 MHz or heat shocked. For each subject, the mean of measurements in 300–600 cells and SD are shown along with p-values for differences compared with sham-exposure by Mann-Whitney U-test. Arrows ↓and ↑ designate direction of effects, decrease or increase, respectively.

a Cases of reported hypersensitivity to electromagnetic fields.

* p < 0.05.

** p < 0.01.

**Table 4:** Changes in γ-H2AX foci in response to 1 hr MW exposure.

	Sham		905 MHz			915 MHz			41°C			43°C		
Subject	γ-H2AX	SD	γ-H2AX	SD	*p*-Value	γ-H2AX	SD	*p*-Value	γ-H2AX	SD	*p*-Value	γ-H2AX	SD	*p*-Value
301^a^	—	—	—	—	—	—	—	—	—	—	—	—	—	—
302	—	—	—	—	—	—	—	—	—	—	—	—	—	—
403^a^	0.91	0.69	7.24↑	1.54	0.00001**	0.10↓	0.25	0.0015**	0.10↓	0.25	0.0105**	0.00↓	0.00	0.0130*
406	1.06	0.57	1.05	1.11	0.4173	0.10↓	0.14	0.0002**	0.52↓	0.40	0.0003**	0.02↓	0.03	0.0003**
501^a^	1.30	1.11	0.09↓	0.08	0.00001**	0.00↓	0.00	0.0007**	0.00↓	0.00	0.0231**	0.02↓	0.02	0.0248*
502	0.06	0.02	0.03	0.04	0.0992	0.00↓	0.00	0.0079**	0.00↓	0.00	0.0002**	0.00↓	0.00	0.0001**
606	0.53	0.32	0.01↓	0.01	0.0079**	0.03↓	0.02	0.0079**	—	—	—	0.00↓	0.00	0.0059**
607^a^	0.36	0.04	0.03↓	0.03	0.0079**	0.04↓	0.02	0.0079**	—	—	—	0.00↓	0.00	0.00001**
707	0.52	0.12	1.22↑	0.38	0.0079**	0.12↓	0.10	0.0079**	0.18↓	0.18	0.0068**	0.68	0.34	0.3402
708^a^	1.34	0.14	1.09	0.34	0.4206	0.18↓	0.05	0.0079**	0.80↓	0.20	0.0011**	0.25↓	0.05	0.00001**
CS	0.54	0.11	0.58	0.65	0.9033	0.06	0.06	0.0808	0.23	0.26	0.1546	0.18	0.34	0.2637
HE	0.98	0.11	2.11	3.45	0.5613	0.08↓	0.08	0.0256*	0.30	0.43	0.0587	0.07↓	0.12	0.0192*
All subjects	0.76	0.11	1.34	2.44	0.5737	0.07↓	0.06	0.0019**	0.27↓	0.32	0.0426*	0.12↓	0.24	0.0029*

—, Not analyzed. Lymphocytes from subjects hypersensitive to EMF (HE; n = 5) and matched controls (CS; n = 5) were exposed to MWs at 905 MHz and 915 MHz or heat shocked. For each subject, mean of measurements in 300–600 cells and SD are shown along with p-values for differences compared with sham by the Mann-Whitney U-test. Arrows ↓and ↑designate direction of effects, decrease or increase, respectively.

a Cases of reported hypersensitivity to electromagnetic fields.

* p < 0.05.

** p < 0.01.

**Table 5:** MW effects on formation of 53BP1 and γ-H2AX foci as analyzed by the Kruskal-Wallis ANOVA by ranks (multiple comparisons of sham, 905 MHz and 915 MHz) in cells from hypersensitive subjects (*n* = 5) and matched controls (*n* = 5).

		*p*-Value	
Subject	No. of images	53BP1	γ-H2AX
301^a^	24	0.0011**	—
302	32	0.00001**	—
403^a^	30	0.00001**	0.00001**
406	27	0.0002**	0.0003**
501^a^	25	0.0002**	0.00001**
502	20	0.0011**	0.0155*
606	16	0.0052**	0.0051**
607^a^	15	0.0034**	0.0075**
707	15	0.0098**	0.0019**
708^a^	15	0.0032**	0.0075**

—, Not analyzed. For each experimental condition, 300–600 cells were analyzed.

a Cases of reported hypersensitivity to EMFs.

* p < 0.05.

** p < 0.01.

**Table 6:** Comparison of MW effects on 53BP1 and γ-H2AX foci at different frequencies, 905 MHz and 915 MHz, in cells from hypersensitive subjects (*n* = 5) and matched controls (*n* = 5) as analyzed by the Mann-Whitney *U*-test.

	No. of images		*p*-Value	
Subject	905 MHz	915 MHz	53BP1	γ-H2AX
301^a^	10	5	0.0007**	0.00001**
302	10	10	0.00001**	0.4173
403^a^	10	10	0.00001**	0.00001**
406	10	10	0.0029**	0.0992
501^a^	10	5	0.0006**	0.0079**
502	10	5	0.0007**	0.0079**
606	5	5	0.1508	0.0079**
607^a^	5	5	0.0317*	0.4206
707	5	5	0.0159*	0.00001**
708^a^	5	5	0.0159*	0.4173

For each experimental condition, 300–600 cells were analyzed.

a Cases of reported hypersensitivity to EMFs.

* p < 0.05.

** p < 0.01.
